# Research progress of gut microbiome and diabetic nephropathy

**DOI:** 10.3389/fmed.2024.1490314

**Published:** 2024-12-13

**Authors:** Chenling Chu, Tapas Ranjan Behera, Ying Huang, Wenhui Qiu, Jiayi Chen, Quanquan Shen

**Affiliations:** ^1^Department of Clinical Medicine, Hangzhou Normal University, Hangzhou, China; ^2^Department of Cancer Biology, Cleveland Clinic, Cleveland, OH, United States; ^3^Department of Public Health and Preventive Medicine, Hangzhou Medical College, Hangzhou, China; ^4^Department of Basic Medicine and Forensic Medicine, Hangzhou Medical College, Hangzhou, China; ^5^Department of Nephrology, Zhejiang Provincial People’s Hospital Bijie Hospital, Bijie, China; ^6^Department of Nephrology, Urology & Nephrology Center, Zhejiang Provincial People’s Hospital (Affiliated People’s Hospital, Hangzhou Medical College), Hangzhou, China

**Keywords:** diabetic nephropathy, gut microbiota, short-chain fatty acids, inflammation, enteric-renal axis, probiotics

## Abstract

Diabetic nephropathy is an important complication of diabetic microvascular injury, and it is also an important cause of end-stage renal disease. Its high prevalence and disability rate significantly impacts patients’ quality of life while imposing substantial social and economic burdens. Gut microbiota affects host metabolism, multiple organ functions, and regulates host health throughout the life cycle. With the rapid development of technology, researchers have found that gut microbiota is closely related to the progression of diabetic kidney disease. This review explores the role of gut microbiome in diabetic nephropathy summarizing proposed mechanisms of progression and focusing on microbial metabolites, intestinal barrier disruption, inflammation, filtration barrier damage and renal fibrosis. This review also examines the mechanism and limitations of current treatments, including drugs, fecal microbiota transplantation, and lifestyle changes, offering new perspectives on prevention and treatment.

## 1 Introduction

According to the data released by the International Diabetes Federation (IDF), there were 537 million diabetes patients in the world in 2021. As the global prevalence of diabetes mellitus (DM) continues to grow, it is projected to rise to 783 million people by 2045 (1). Type 2 diabetes mellitus (T2DM) accounts for more than 90% of the global incidence (2). During the prolonged course of diabetes leading to chronic kidney disease (CKD), 30 to 40% of patients develop signs of kidney injury, making diabetes a major driver of kidney failure (3). The various causes of CKD in diabetic patients include diabetic nephropathy (DN), ischemic kidney disease associated with vascular disease, nephrosclerosis due to hypertension, and kidney disease unrelated to diabetes (4). Studies of DN reveal that its progression is closely related to the duration of diabetes, hypertension, blood sugar control, and smoking (5). The natural history of disease suggest that within a span of 5 years or more, the patient’s proteinuria worsens progressively from normal to microproteinuria, and the glomerular filtration rate (GFR) gradually decreases to less than 60 mL/min/1.73 m2, eventually leading to end-stage renal disease (ESRD) (6, 7). In recent years, some researchers have found that gut microbiota, owing to its richness and diversity, has an impact on nutrient utilization and bioactive metabolites in the body during the development of diabetic kidney disease (DKD). After in-depth research, it is currently believed that the intestinal flora of DKD patients is disordered, and the metabolic derivatives of the flora accumulate in the body, causing a series of reactions such as glomerular filtration barrier damage in the kidney, renal interstitial inflammation, oxidative stress, and ultimately leading to the deterioration of renal function. The constituent and diversity of gut microbiota varies significantly among diabetic individuals due to the influence of obesity, insulin resistance, dyslipidemia and other factors, which in turn participate in the host’s dietary intake, energy acquisition, consumption, and fat storage (8). The bidirectional functional relationship between the colon and the kidney is called the colon-kidney axis. In CKD, a decreased clearance of renal metabolites further enhances the absorption of uremic toxins produced by the gut microbiota promoting the progress to ESRD (9). The variation in gut microbiota among individuals are affected by disease, dietary intake and derived metabolites, and could be an adaptation of needs of the body to the environmental challenges (10).

This review aims to summarize the current status of research on the influence of gut microbiome in the progression of DN, and to discuss the prevention or delay of CKD progression in diabetic patients that could be achieved by modulating the gut microbiome through lifestyle changes and drug intervention.

## 2 Epidemiology and histopathology of DN

According to data released by IDF, the incident cases of CKD as a result of T2DM worldwide have increased by 74% from 1990 to 2017, with the highest incidence of ESRD was found in some Asian countries and the United States (1). As the primary organ for accumulation and clearing advanced glycation end products (AGES) in diabetes mellitus, the kidney is the earliest organ for AGES-mediated damage (11). From the histopathological perspective, kidneys in DN demonstrate extracellular matrix (ECM) deposition, thickening of the glomerular basement membrane, tubular atrophy, and cell proliferation leading to interstitial fibrosis and glomerular sclerosis (12). These histopathological changes in DN are not only related to diabetic microangiopathy, but are also affected by large vascular disease, aging, atherosclerosis, hypertension and acute kidney injury (7). During the development of DN, the hyaline arteriolosclerosis of the glomerular afferent and efferent arteries leads to glomerular hyperfiltration and increased albumin excretion, which can lead to diffuse diabetic glomerulosclerosis and typical nodular glomerulosclerosis (Kimmelstiel-Wilson nodules) in the late stage of DKD (13).

## 3 Gut microbiota imbalance as a novel diagnostic and therapeutic biomarker for DKD

In many cases, DKD is a clinical diagnosis. Currently the kidney puncture biopsy is the gold standard for diagnosing DKD. Mazzucco et al. (4) conducted kidney biopsy on 393 T2DM patients, and more patients in the unlimited needle biopsy policy group were found to have other glomerulonephritis, indicating that the lack of standardized diagnosis has an impact on the epidemiological data of DKD. Therefore, the diagnosis of DKD by histopathology is more definitive than that made only from the history and test criteria. Therefore, the diagnosis of DKD is based on the 2020 American Diabetes Association (ADA) guidelines for the diagnosis of DKD (14). The ADA and Kidney Disease: Improving Global Outcomes (KDIGO) group recently published the relevant guidelines. All patients diagnosed as having diabetes should have their renal function and albuminuria examined and undergo annual reexaminations (15).

In the process of clinical application of urinary albumin and eGFR to evaluate DKD, it is found that about 30% of DKD patients have no albuminuria (16). With the expanding incidence of DKD and the limitations of changes in urinary albumin excretion rate and eGFR in predicting the progression of DKD, early diagnosis of DKD has become an urgent global issue. The large and complex microbial community in the human gut interacts with human diseases, and its metabolites can show the relationship between intestinal flora and diseases more directly. In the fecal sequencing results of DM, early and late DKD patients, it was found that the disorder of gut microbiota in early DKD patients was particularly prominent, and there were different intestinal microbial characteristics at different stages of DKD. Among them, Agathobacter has been proposed as the most meaningful gut bacterial biomarker to distinguish DKD stages (17). A large number of studies have suggested that metabolic derivatives of gut microbiota play an important role in the pathogenesis of DKD. Therefore, many researchers have applied metabolomics to analyze the role of metabolites of gut microbiota in the early diagnosis of DKD (18). Balint et al. (19) found that arginine, dimethylarginine, hiproic acid, indolyl sulfate(IS), butylcarnitine and sorbitol have specific trends in the serum of patients with early DKD, which can indicate early metabolic disorders in patients with DKD. And acryloyl sulfate in urine may indicate early DKD. Ng et al. (20) found oxalic acid, octanol, phosphoric acid, creatinine, phenyl amide, 3,5-dimethoxy mandelic amide, IS, N-acetyl glutamine and various uremic toxins in the urine of T2DM patients without proteinuria, which can predict the decline of eGFR. In addition, some studies have shown that serum cystatin C (Cys C) is more sensitive than urinary albumin excretion rate and serum creatinine and plays an important role in the early diagnosis of DKD (21). These metabolic derivatives of gut microbiota can be used as early candidate biomarkers for DKD, suggesting metabolic disorders before the occurrence of albuminuria or decreased eGFR in patients with DKD. The development of interventions targeting specific metabolites or metabolic pathways provides directions for the prevention and treatment of DKD.

## 4 Mechanisms of DKD

Mechanisms of diabetes-induced CKD is multifactorial. It involves inflammation, renal oxidative stress, autophagy among others. The structural changes including that of endothelial cells and podocytes could be attributed to the high glucose microenvironment. It is now believed that inflammation and immune responses drive disease progression in the early stages of DKD (22). It is noted that about 80% of T2DM patients are overweight or obese; and obesity, especially visceral obesity, can lead to inflammation, leaving the body in a low-grade inflammation state (23). The high glucose environment in diabetic patients can promote the intracellular activation of polyols and protein kinase C (PKC) pathway to form AGES, as well as glomerular hypertension induced by glomerular hyperfiltration, and then promote the occurrence of DN inflammation through downstream inflammatory factors (24). Inflammatory cytokines, such as interleukin (IL)-1, IL-6, IL-18, and tumor necrosis factor-*α* (TNF-α), can be synthesized by different renal cells that plays a role in increasing the permeability of vascular endothelial cells, promoting the induction and differentiation of inflammatory cells, and activating apoptosis, thus promoting the development of DKD (25). The oxidative stress response of the kidney is enhanced under the mediation of NADPH oxidase (NOX), leading to the generation of excessive reactive oxygen species (ROS) which in turn leads to significant tissue damage by promoting lipid peroxidation, DNA damage, protein modification, and mitochondrial dysfunction (26). Autophagy is one of the protective mechanisms of the kidney, as it can recover energy from nutrient depletion and remove damaged organelles and cytotoxic proteins during stress (27). Fang et al. (28) found that in prolonged high glucose environment, the podocyte autophagy level medicated thorough the endoplasmic reticulum stress exhaustion, was decreased significantly leading to damage to the glomerular filtration barrier thus promoting deterioration of DKD.

### 4.1 Possible mechanisms of DKD involving intestinal microflora

In recent years, in addition to relatively better understood mechanisms such as inflammation and oxidative stress, some researchers have found a close relationship between DKD and gut microbiota. The gut microbiota contains over 100 trillion microorganisms, over 1,000 different species of bacteria, which have been shown to be involved in physiological and pathophysiological host processes, with two-thirds of the gut microbiota being individual-specific (29). The disturbance of gut microflora and the change of gut microflora structure can lead to the changes and disorders of immune inflammation, body metabolism, and signal transduction, which has shown to affect the inflammatory response of the intestinal tract, liver, kidney, brain and other organs (30). Gut microbiota adapts to the microenvironment of the body and can change due to proteinuria, inflammation, T2DM, hypertension and other factors, especially closely related to insulin resistance and lipid metabolism disorders (31). The gut microbiota of the healthy people is mainly composed of Firmicutes and Bacteroidetes, followed by actinobacteria and verrucoides (32). Comparing the gut microbiota of healthy control group, DM, DKD, and non-diabetic nephropathy (NDKD), no *α* diversity was observed between DKD and DM and NDKD, respectively. The abundance of gut microorganisms in DKD group however was lower than that in healthy control group. In other findings, the phylum Actinobacteria, Hungatella, Escherichia and Lactobacillus were enriched in the DKD group. Butyricicoccus, Faecalibacterium and Lachnospira genera were depleted in DKD. Among them, the enrichment of Hungatella and Escherichia is the characteristic of the change in gut microflora of DKD (33). There were significant differences in gut microflora sequencing in ESRD induced by DKD and NDKD. The strains with the largest differences included Oscillibacter, Bilophila and UBA1819, which provided a basis for further exploration of the microflora related to the progression of DKD (34). It’s proposed that the imbalance of gut microbiota in DN patients promotes various cellula processes such as citrate cycling, base resection and repair, histidine metabolism, lipoic acid metabolism and bile acid biosynthesis in the body, while selenium metabolism and branched-chain amino acid biosynthesis were observed to be decreased (35). It can be seen from studies that the gut microbiota is closely related to the development process of DKD, and the structure, number and function of the gut microbiota undergoes significant changes during this process, but its regulatory mechanisms are complex and diverse, and still not fully understood.

In the process of metabolism and immune inflammation, intestinal microbiota produces related metabolites, such as inflammatory factors, enterotoxin-derived toxins, etc. Mediated by these metabolites, the intestinal immune barrier is destroyed, leading to “intestinal leakage,” and keeping the body in a state of chronic low-degree inflammation (10) (Table 1).

**Table 1:** Mechanisms of gut metabolites.

Name	Mechanism	Refs
LPS	LPS bind to TLR4, induce GBPs expression, and mediate caspase-11 pathway to participate in renal tubulointerstitial inflammation. LPS combined with TLR4, activate the NF-kB signaling pathways. They enhance NLRP3 expression in renal epithelial cells, and participate in the kidney inflammation and fibrosis	(36, 40).
TMAO	Increased mitochondrial ROS generation, participate in activated mROS—NLRP3 axis to adjust the body’s inflammatory response, DKD white proteinuria and renal tubular interstitial fibrosis	(48)
IS	Activation of AhR in mouse podocytes and promotion of the expression of vimentin and AhR increase the expression of cytokines and chemokines in podocytes, which eventually lead to glomerular filtration barrier damage, mediating renal tubular cell apoptosis, necrosis, interstitial injury, and promoting renal fibrosis	(49)
SCFA	Inhibition of NF-kB activation and islet β-cell inflammation can reduce proteinuria, stabilize glomerular basement membrane podocytes, and reduce glomerulosclerosis and renal inflammation SCFA reduction leads to inhibition of intestinal histone deacetylation, weakens histone post-translational modification, and promotes and participates in EMT of renal tubular epithelial cells	(60, 62)
MAVS	Activate the downstream transcription factors IRF3 and NF-kB, MAVS knockout mice intestinal generated *Klebsiella oxytoca* and IL—17, stimulate the intestinal epithelial cells inflammation and injury of renal tubular epithelial cells generated molecules—1, increases intestinal permeability, leading to increased glomerular and renal tubular damage	(66)
BA	BA metabolic disorders lead to cholestasis and intestinal inflammation, reduce the expression and receptor activity of FXR/TGR5, reduce renal protective function, increase proteinuria, and promote podocyte injury, lipid accumulation, and fibrosis.	(70)
HA	HA can promote the progression of renal fibrosis, disrupt NRF2-KEAP1-CUL3 to break the redox balance and increase the expression of fibrosis-related genes in CKD.	(75)
H2S	H2S is produced by fermentation of sulfate and cysteine, which inhibits oxidative stress, regulates GFR, and improves hemodynamics of DN	(80)

LPS, lipopolysaccharide; TLR4, Toll-like receptor 4; GBPs, guanylate binding protein; NF-kB, nuclear factor kappa-B; NLRP3, nucleotide-binding oligomeric domain-like receptor protein; TMAO, Trimethylamine-N-oxide; ROS, reactive oxygen species; IS, indoline sulfate; DKD, diabetic kidney disease; AhR, aryl hydrocarbon receptor; SCFA, short-chain fatty acids; EMT, epithelial-mesenchymal transition; MAVS, mitochondrial antiviral signaling protein; IRF3, interferon regulatory factor; IL, Interleukin; GFR, glomerular filtration rate; DN, Diabetic nephropathy; BA, bile acid, HA, hippuric acid.

#### 4.1.1 Effect of outer membrane vesicles and lipopolysaccharide in DKD

Among the four intestinal gram-negative bacterial phyla (Bacteroidetes, Proteobacteria, Fusobacteria and Verrucomicrobia) of T2DM-CKD, the relative abundance of Proteobacteria, Verrucomicrobia and Fusobacteria increases significantly (36). Outer membrane vesicles (OMVs) play an important role in kidney damage. OMVs are extracellular vesicles (EVs) released by Gram-negative bacteria and contain a variety of components, including abundant lipopolysaccharide (LPS) (37). LPS, also known to be endotoxins, are an important component of the outer membrane of the cell wall of Gram-negative bacteria and can stimulate immune cells, such as macrophages and endothelial cells, to synthesize and secrete inflammatory factors and mediate brain, kidney and lung tissue damage (38).

**Figure 1:**
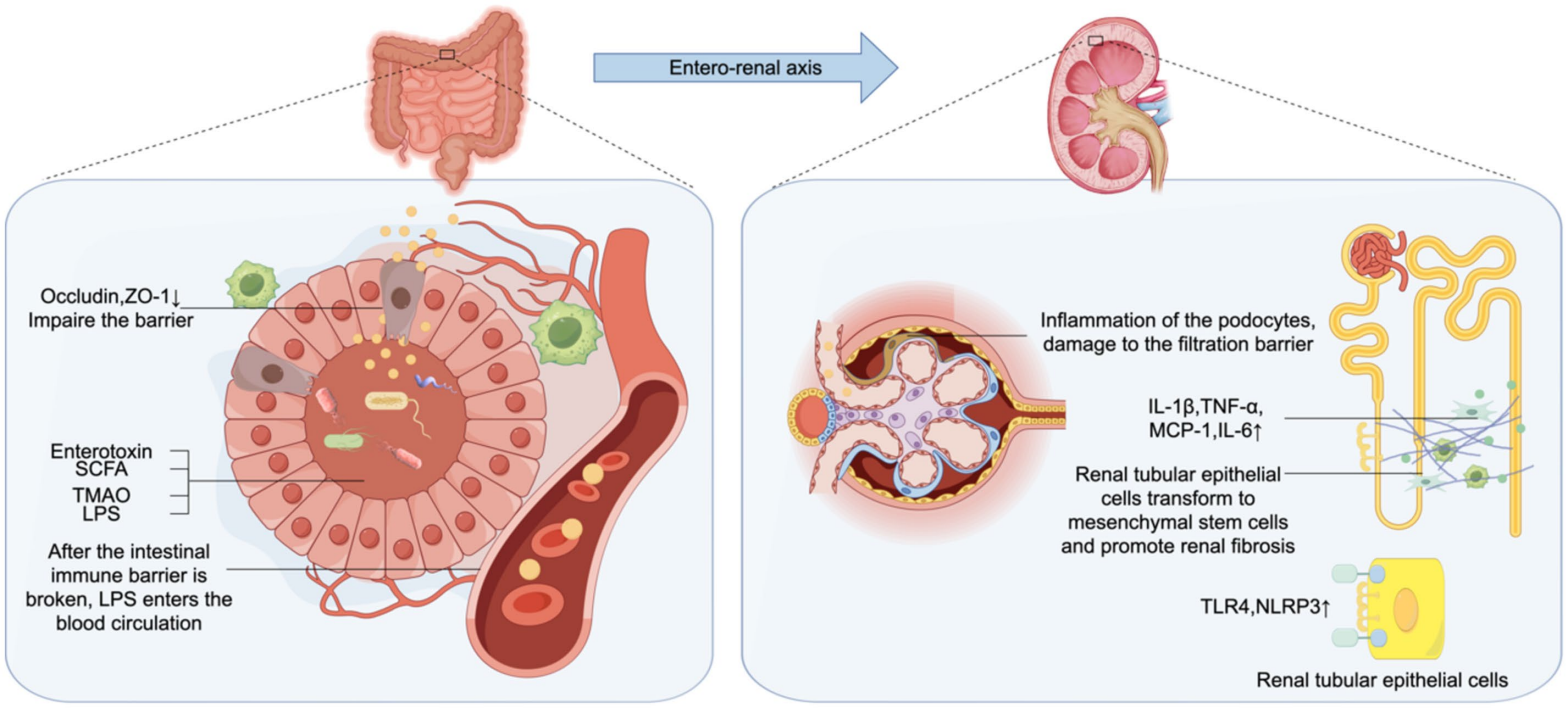
Gut-kidney axis. The increase of outer membrane vesicles (OMV) produced by Gram-negative bacteria, OMV contains abundant lipopolysaccharide (LPS), which reduces the intestinal tight junction related proteins occludin and zonula occludens-1 (ZO-1), and damages intestinal barrier. These are transferred to the renal tubulointers space through blood circulation, leading to enhancced expression of inflammatory factors such as interleukin (IL)-1β, tumor necrosis factor (TNF)-*α*, monocyte chemotactic protein (MCP)-1 and IL-6 in renal tubular epithelial cells through the Caspase-11 pathway. LPS binds to Toll-like receptor (TLR)4, up-regulates the expression of TLR4 and nucleotide-binding oligomeric domain-like receptor protein (NLRP)3 and promotes renal fibrosis. Enterogenous endotoxin damage of intestinal epithelial cells, the damage of glomerular filtration barrier, lead to increased proteinuria.

EVs produced by gut microorganisms can get transferred to distant organs and tissues, inducing inflammation and dysfunction of multi-tissue organs, and mediate tubulointerstitial inflammation in DKD (39). When diabetes leads to gut microbiota disturbance, the abundance of gram-negative bacteria in the gut increases, and the production of OMV increases, leading to the increase of LPS. High levels of serum LPS are significantly correlated with the accumulation of inflammatory factors such as C-reactive protein (CRP), TNF-*α*, and IL-6 in DKD patients, and with the progression of DKD (36, 40). Chen et al. (41) found that the gut microbiota of diabetic mice produces increased OMV, which reduces intestinal tight junction-related proteins occludin and occludens-1 (ZO-1), mediating intestinal integrity impairment. OMV gets transferred to renal tubulointerstitial regions, where they promote the expression of inflammatory factors such as IL-1β, TNF-α, monocyte chemotactic protein (MCP)-1 and IL-6 in the renal tubulointerstitial cells through the Caspase-11 pathway, thus inducing tubulointerstitial inflammation. It has been reported that Caspase-11 is also involved in the inflammation and injury of diabetic mouse podocytes, inducing kidney injury. In exploring the mechanism of action of OMVs, it was found that the binding of LPS released by OMVs to Toll-like receptor (TLR)4 is the initial and necessary process for the subsequent induction of inflammation. It was shown that blocking of the expression of TLR4 receptor gene or the involved signaling pathways led to alleviation of the LPS-induced inflammatory response and insulin resistance, thus effecting as attenuation of renal fibrosis of CKD is alleviated (42, 43). LPS induces the expression of guanylate-binding proteins (GBPs) through interferon-*β* / interferon regulatory factor/type I interferon pathway under the action of adaptor containing TIR domain, which can mediate the DKD renal tubulointerstitial inflammation of caspase-11 pathway (41). Studies have shown that TLR4 expression is up-regulated in human DKD renal tissue, and gut microbiota-derived circulating LPS binds to TLR4, activates the nuclear factor kappa-B (NF-κB) signaling pathway, and up-regulates the expression of nucleotide-binding oligomeric domain-like receptor protein (NLRP) 3 in renal epithelial cells mediated by TNF-*α* and IL-12. It mediates the production of inflammatory cytokines and apoptosis of renal tubular epithelial cells. In addition, NLRP3 can promote renal fibrosis through the Transforming Growth Factor (TGF)-*β*-Smad signaling pathway (42, 44) (Figure 1). In DKD populations, LPS mediates albuminuria by up regulating the expression of CD80 in podocytes, and serum LPS activity is an independent risk factor for the development of DKD (40). In DKD, gut microbiota-derived OMVs activate TLR4 receptor, Caspase-11 pathway and NF-κB pathway by releasing LPS, and promote the accumulation of inflammatory factors in the renal tubulointerstitium.

#### 4.1.2 Adverse effect of enterogenous toxins in DKD

During the progess of CKD to ESRD, electrolytes, hormones and uremic solutes (URS) accumulate in the body and interfere with the biological functions of the body. The protein-bound URS could not be completely removed even after renal replacement therapy (45). Quantitative tests on metabolites of gut microbiota in hte diabetic population found that free amino acid arginine and its metabolites, uremic toxins, acylcarnitine and sorbitol were correlated with the severity of DKD albuminuria (19). In early CKD patients, arginine and asymmetric dimethylarginine (ADMA) can reduce renal microvasodilation, impair normal glycolysis, regulate oxidative stress, and mediate the generation of reactive oxygen species (ROS), to maintain chronic inflammation in the body and generate renal albuminuria (19).

Diabetes disrupts the gut microbiota, resulting in uremic toxins such as urea nitrogen (BUN), serum creatine (SCR), IS, phenyl sulfate (PS), and Trimethylamine-N-oxide (TMAO) concentration (19). As a low molecular weight uremic toxin, TMAO is positively correlated with insulin resistance, glucose tolerance, and the severity of diabetic retinopathy (46). Xu et al. (47) found that DKD patients’ serum TMAO level increased significantly, and the level of BUN and SCR were positively correlated. Under the influence of gut microbiota, the body increases TMAO levels by metabolizing choline, betaine, L-carnitine, and trimethylamine (TMA). It was seen that TMAO has a significant role in the development of DKD. TMAO increased the generation of mitochondrial reactive oxygen species and participated in the activation of mROS-NLRP3 axis to regulate the inflammatory response of the body, albuminuria of DKD and renal tubulointerstitial fibrosis (48). As a transcriptional regulator, IS mediates renal interstitial and vascular injury. IS can affect through the cellular mechanism in various ways such as to activate the aryl hydrocarbon receptor (AhR) of mouse podocytes and promote the expression of vimentin and AhR. It increases the expression of cytokines and chemokines in podocytes while decreasing the expression of actin cytoskeleton protein and collagen, thus affecting the function of podocytes, and eventually leading to the damage of glomerular filtration barrier and the formation of albuminuria. At the same time, IS can mediate apoptosis and necrosis of renal tubule cells and interstitial injury and promote renal fibrosis (49). In conclusion, intestinal microorganisms can produce a variety of enterotoxins into the circulatory system after the destruction of the intestinal immune barrier, causing or aggravating DKD through various mechanisms such as mediating inflammation, oxidative stress, and damaging the filtration barrier of the kidney.

#### 4.1.3 Role of short-chain fatty acids in DKD

**Figure 2:**
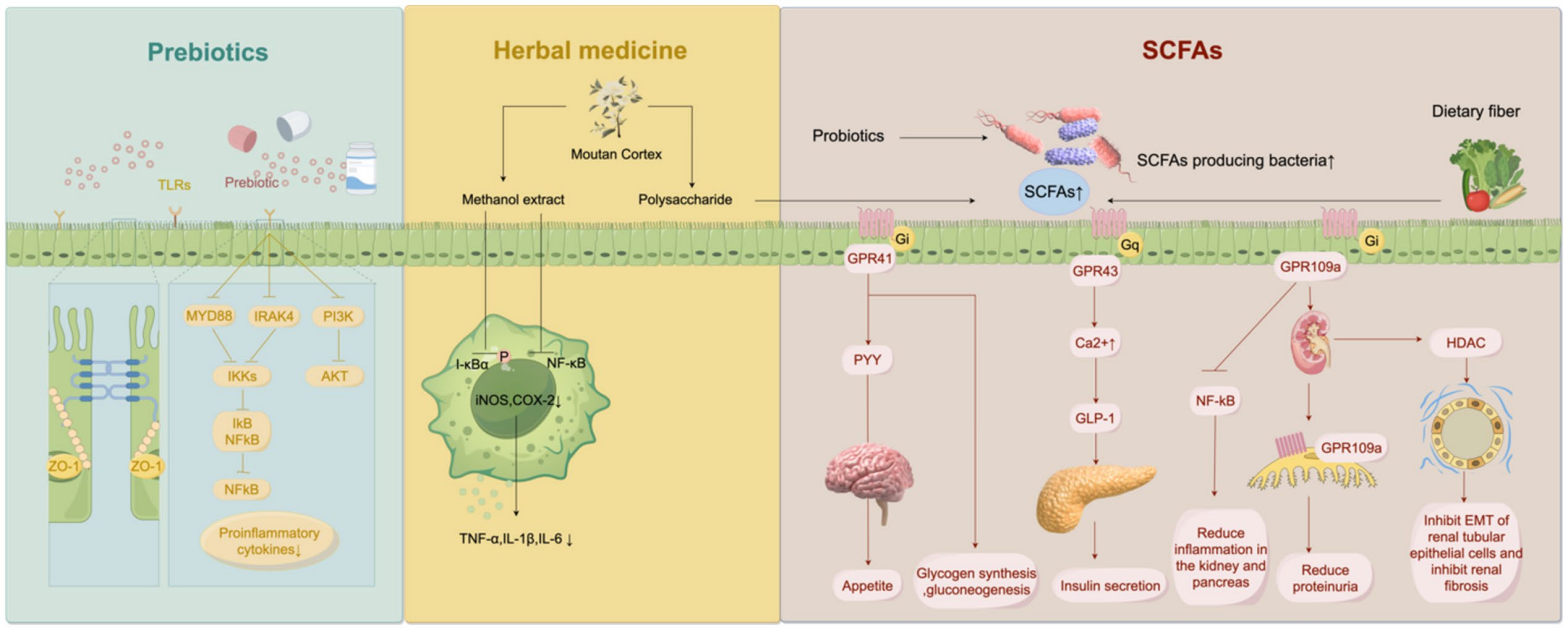
Multiple drug treatments that interfere with the gut microbiota. Prebiotics inhibites Toll-like receptor/nuclear factor kappa-B (TLR/NF-κB) signaling pathway and phosphatidylinositol-3-kinase-protein Kinase B (PI3K-AKT) pathway. They reduce the production of pro-inflammatory factors, and repaired the tight junction between intestinal epithelium. Methanolic extract of Moutan Cortex (MC) reduces Inhibitor-κBα (I-κBα) phosphorylation and NF-κB activation in macrophages by inhibiting lipopolysaccharide (LPS), thereby inhibiting inducible nitric oxide synthase (iNOS) and Cyclooxygenase-2 (COX-2) expression, and decreasing concentrations of tumor necrosis factor-α (TNF-α), interleukin (IL)-1β, and IL-6. MC polysaccharides can reshape the intestinal flora to produce more short-chain fatty acids (SCFA) in diabetic kidney disease (DKD) rat model, and reduce serum inflammatory factors and alleviate kidney injury in the process.

The level of metabolite short-chain fatty acids (SCFA) is decreased after gut microbiota imbalance. SCFAs are the end product of intestinal microbiota fermentation of dietary polysaccharides, including butyrate, acetate, propionate, valerate and isocaptanoic acid. In the intestinal microbiome, *Faecalibacterium prausnitzii* (*F. prausnitzii*) is one of the most abundant bacteria in the human gut, and its relative abundance decreases in DKD. The decrease of F. prusnitzII leads to the decrease of SCFA production, affects energy absorption, and leads to intestinal anti-inflammatory, immunomodulatory dysfunction and kidney injury (50). SCFA can mediate cell signaling pathways, participate in cell proliferation, apoptosis and histone acetylation. SCFA is currently a new target for the treatment of systemic inflammatory, immune and metabolic diseases associated with glucose tolerance, obesity and insulin resistance (51). Rectal and intravenous administration of acetate had no effect on the levels of free fatty acids and insulin, but increased glucose and glucagon levels, and decreased TNF in plasma. Polypeptide YY was increased in the rectal administration group (52). After comparing the clinical data of the healthy group and the hyperinsulinemia group, it was found that the free fatty acids in the healthy group were decreased, and the degree of decrease in free fatty acids was negatively correlated with the insulin resistance index. Thus, acetate can inhibit lipolysis and improve insulin resistance (53). When exploring the mechanism of SCFA, it was found that the basic level of glucagon-like peptide (GLP)-1 in mice with knocked out G protein-coupled receptor (GPR)-43 was reduced by nearly 43%, and the levels of peptide YY and GLP-1 in the gut were still decreased after propionate administration (54). It is concluded that propionate in SCFA mediates GPR-43 signal transduction to promote the synthesis of peptide YY and GLP-1, and participates in glucose regulation. Lin et al. (55) found that SCFA differentially activated GPR. Similarly, propionic acid activates GPR41 and GPR43, acetic acid activates GPR43, and butyric acid activates GPR41, although the activation receptors are not the same, all of which can reduce diet-induced obesity and insulin resistance. When studying the involvement of SCFA in kidney injury, it was found that SCFA-activated Olfr-78, GPR-43, GPR-41 and GPR-109A were all expressed in the kidney (56, 57). Among them, GPR-109A can inhibit NF-kB activation and pancreatic *β* cell inflammation, and the increased expression of GPR-109A in renal injury models can alleviate proteinuria, stabilize glomerular basement membrane podocytes, and alleviate glomerular sclerosis and kidney inflammation (58) (Figure 2). Activation of Olfr-78 expressed by the kidney can activate the renin-angiotensin system (RAS), and angiotensin II (Ang II) can lead to renal vasoconstriction and glomerular hypertension (59). The morphology of podocytes and glomerular endothelial cells changed under the action of Ang II, and the deposition of the extracellular matrix accelerated the progression of DN (60). In addition, reduced SCFA produced by the microbiota leads to inhibition of histone deacetylation and weakened post-translational modification of histones in the gut, which promotes epithelial-mesenchymal transformation (EMT) of renal tubular epithelial cells and participates in renal fibrosis of DN (61). Therefore, some researchers believe that the decrease in bacteria-producing SCFA can promote the inflammation of the kidney and multiple other organs and can be ascribed to the deterioration of renal function in DN (62). In the future, gut microbiota involved in SCFA anabolism, such as F. Prusnitzii, could be used for clinical management and treatment.

#### 4.1.4 Impact of mitochondrial antiviral signaling protein on DKD

The mammalian innate immune system uses pattern recognition receptors (PRRs), including TLRs, NOD-like receptors (NLRs), and retinoid-induced gene-1 (RIG-I) like receptors (RLRs), to participate in immune recognition and activate inflammatory cytokines (63). RLRs recognizes the 5 ‘-triphosphate and double-stranded structure of viral RNA and can induce the production of interferon (IFN) -*α*/*β* after activation. RIG-I recruits mitochondrial antiviral signaling protein (MAVS), a mitochondria-targeted antiviral protein that activates downstream interferon regulatory factor (IRF3) and NF-κB, which play an important role in the body’s innate immune response (64). After constructing the MAVS knockout diabetic nephrotic mouse (MAVS−/−) model, Linh et al. (65) compared the results of kidney and intestinal damage, bacterial translocation and other aspects, and found that the gut of MAVS−/− produced more *Klebsiella oxytoca* and IL-17. It can stimulate the inflammation of intestinal epithelial cells and produce kidney injury molecule-1 (KIM-1) in tubular epithelial cells, increase intestinal permeability, and aggravate the damage of glomeruli and renal tubules. It has been found in studies of many diseases that a lack of MAVS can cause changes in intestinal bacteria, damage the intestinal barrier, and destroy epithelial integrity (66). In the current study, modulating the intestinal barrier by controlling MAVS signaling is a potential therapeutic target for delaying DKD kidney damage.

#### 4.1.5 Effect of other metabolites on DKD

Some researchers have paid attention to other metabolites and actively searched for the mechanism by which metabolites cause or accelerate the progression of DKD. Researchers found that serum free bile acid (BA) levels were significantly lower in patients with DKD, and their levels were significantly correlated with increased proteinuria and decreased eGFR (67, 68). Further exploration of the mechanism of BA in DKD metabolism found that Firmicutes, Bacteroidetes, Lactobacillus, Bifidobacterium and Clostridium can convert intestinal BA into secondary bile acids and affect lipid metabolism through enterohepatic circulation (EHC) (69). BA binds to Farnisidium X receptor (FXR) and Takeda G protein-coupled receptor 5 (TGR5) to inhibit renal injury processes such as increased proteinuria, podocellular injury, lipid accumulation, and fibrosis (70). However, the decrease of Bifidobacterium, Firmicutes and Actinobacteria in DKD patients leads to decreased enzyme activity of BA bioconversion, cholestasis and intestinal inflammation (71). Dysregulation of BA metabolism further affects FXR/TGR5 expression and receptor activity. FXR and TGR5 expression levels are decreased in DN, aging and calorie-restricted kidneys, and their renal protection mechanisms are weakened (72). INY-767(FXR/TGR5 double agonist), INT-777(selective TGR5 agonist), and OCA (selective FXR agonist) can improve proteinuria, alleviate podocyte injury, inhibit renal fatty acid and cholesterol metabolism, and reduce fibrosis-related protein production in diabetic and high-fat obese mice (72). When FXR is knocked out, DKD is aggravated and accompanied by lipid metabolism disorders (73). The synergistic protective effect of FXR and TGR5 on renal inflammation, oxidative stress, albuminuria and fibrosis is of reference value for the treatment of diabetes and obesogenic nephropathy.

Zhang et al. found that 11 types of gut microbiota in DKD patients were associated with 192 serum metabolites. Metabolites produced by microbiota through the phenylalanine metabolic pathway, such as hippuric acid (HA), L-(−)-3-phenylactic acid, trans-3-hydroxy-cinnamate, dihydro-3-coumaric acid, which can promote the development of DKD. However, L-tryptophan, a metabolite of the tryptophan pathway, can alleviate kidney injury (74). As an important product of phenylalanine metabolism of gut microbiota, HA can promote the progression of renal fibrosis by disrupting NRF2-KEAP1-CUL3 to break the redox balance and increase the expression of fibrosis-related genes in CKD (75).

In addition, sulfates and cysteine reduce hydrogen sulfide (H2S), the metabolic product generated after bacterial fermentation in the intestine, which is involved in regulating GFR, improving the hemodynamics of DN, and inhibiting oxidative stress (76). Sodium hydrosulfide (NaHS) reduces inflammatory cell infiltration, tubule atrophy and interstitial fibrosis by inhibiting NF-κB activity, and significantly improves renal function in DN rats (77).

Diabetes mediates the disturbance of the gut microbiota, and its metabolites disrupt the intestinal immune barrier, leading to renal interstitial inflammation, destruction of the glomerular filtration barrier and renal fibrosis through mediating multi-pathway signal transduction pathways. Some researchers try to control the progression of DKD by blocking the participating signaling pathway, but the current state of research has limitation in translation into clinical applications.

## 5 Mechanisms for treating DKD

The conventional treatment of DN is mainly through lifestyle intervention, which can effectively delay the progress of DKD by controlling blood sugar, stabilizing the blood pressure, and reducing albuminuria. By regulating the gut-kidney axis, gut microbiota plays a vital role in the chronic course of CKD caused by diabetes (78). Various clinical trials have found that probiotics, prebiotics, traditional Chinese medicine, fecal microbiota transplantation and other ways to improve the intestinal microecology can prevent and delay the deterioration of kidney function (Figure 2).

### 5.1 Dietary fiber and prebiotics

Dietary fiber is not digested or absorbed by the body. The bacterial fermentation occurs in the gastrointestinal tract to affect gut microbes. Different types of fiber contained in food have specific fermentability, solubility, and viscosity, which affect fermentation and biological metabolism. For example, high solubility and viscosity can control blood sugar and lower cholesterol levels, while insoluble fibers are not easily fermented by intestinal microorganisms, improving intestinal transport rates (79, 80). Li et al. (81) proved that a high-fiber diet contributes to the recovery of gut microbiota, increases the production of SCFA, mediates GPR43 or GPR109A, reduces the expression of inflammatory factors, and reduces the incidence of kidney injury in diabetic mice.

The unique dietary fibers in the prebiotics are inactive ingredients in food that are beneficial to host health and participate in regulating body immunity and maintaining gastrointestinal function, including inulin, dextrose and lactulose (82). Currently, recovery of bifidobacterium and Lactobacillus, production of SCFA-related bacteria *Faecalibacterium prausnitzii* and mucus-degradation bacterium *Akkermansia muciniphila* are the main targets of prebiotic feeding. These bacteria are closely related to reducing inflammation and maintaining the intestinal immune barrier (83). In animal models, prebiotics can maintain the intestinal immune barrier by protecting the integrity of the agglutinin of intestinal epithelial cells, reducing enterogenic LPS entering the blood circulation, and reducing the level of inflammatory cytokines (84). In addition, prebiotics control inflammation and metabolic disorders in obesity and diabetes by enhancing GLP-1/2 expression (85). It has been proved that supplementation of prebiotics such as inulin-type fructose (ITFs) and fructooligosaccharides can protect kidney function through the regulation of gut microbiota (86). The types and properties of dietary fiber and prebiotics are different, and the specific mechanism of their action in the gut still needs to be explored further.

The safety and effectiveness of dietary fiber and prebiotics make them great interventions for clinical application. Jia et al. (87) clinically found that increased dietary fiber intake in U.S. adults with type 2 diabetes may reduce the incidence of DKD. Some researchers have proposed that a vegetarian diet has a protective effect on the kidney of DKD when calculating the effect of dietary fiber intake on the kidney in DM patients (88). Several prospective cohort studies have also demonstrated that dietary fiber intake can reduce the risk of T2DM (89). Dietary regulation is an important principle in the treatment of diabetes. Lifestyle that increases dietary fiber diet has also shown protective effects on renal function in DKD patients. In the clinical study of prebiotics, prebiotics can reduce the levels of CRP, IL-6, TNF-*α*, SCr and BUN in the serum of patients with T2DM nephropathy stage IV and increase eGFR. In addition, the incidence of complications and the rate of secondary hospitalization were reduced in patients using prebiotics (90). At present, the use of prebiotics in DKD patients has been found to reduce microinflammation, promote toxin excretion, and delay the deterioration of renal function.

### 5.2 Probiotics

Clinical and experimental data show that gut microbiota is important in mediating kidney injury in diabetic patients. Direct oral administration of sufficient probiotics has positive protective effects on intestinal homeostasis, intestinal immunity, and kidney, among which the most widely used probiotics include lactobacillus and bifidobacterium (91). Probiotics have various mechanisms of action, which can not only increase the survival rate of intestinal cells when gut microbiota is disturbed and immune barrier is damaged but also up-regulate the expression of ZO-1 to increase the tight connection between intestinal epithelial cells, to avoid large amounts of intestinal toxins entering the blood circulation after the intestinal barrier is broken (92). The probiotic *Lactobacillus plantarum* A7 has been found in studies to reduce albuminuria, creatinine, and GFR in patients with DN by reducing inflammation and improving oxidative stress levels (93). In the process of controlling blood sugar in DN, the mixture of *Lactobacillus acidophilus*, bifidobacterium, Lactobacillus multiplex and *Lactobacillus fermentum* has been confirmed to improve insulin sensitivity and thus reduce blood sugar (94). Probiotics need to exert physiological effects after colonizing the gastrointestinal tract, so their effects are affected by the environment of the individual digestive tract. Supplementation of probiotics in elderly patients with DKD is more beneficial to control blood glucose and blood lipids, promote the excretion of SCr and BUN, reduce the level of 24-h urinary protein, and protect renal function. At the same time, probiotics can inhibit inflammatory factors and promote the production of antioxidant factors (95). Probiotics play a unique advantage in the treatment of patients with DKD by regulating gut microflora.

### 5.3 Herbal medicine

In clinical and animal studies, herbs have been shown to protect the kidneys and regulate blood sugar. Zhang et al. (96) evaluated the effectiveness and safety of Chinese herbal medicine in the DKD population through systematic review and meta-analysis and proved that Chinese herbal medicine can significantly improve renal function and proteinuria, and the incidence of adverse events is very low. Moutan Cortex (MC), as an anti-inflammatory and antioxidant Chinese medicine, has extensive pharmacological activities in diabetes control and cardiovascular protection. The methanol extract of MC inhibits the expression of inducible nitric oxide synthase (iNOS) and Cyclooxygenase (COX)-2 by inhibiting the phosphorylation of Inhibitor-κB*α*(I-κBα) by LPS and the activation of NF-κB in macrophages, leading to decrease in the concentrations of TNF-α, IL-1β and IL-6, which confirms its anti-inflammatory effect (97). In the DKD rat model, MC polysaccharide reshaped the gut microflora in the body to produce more SCFA, and in the process reduced the serum inflammatory factors of rats and alleviated kidney injury (98). Another plant drug, *Morus alba* L(ML), works in DKD rats by down-regulating NEFA signaling and restoring phyla Bacteroidetes and Proteobacteria and class Clostridia, which are associated with insulin resistance. Improved insulin resistance and lipid levels reduce urinary protein levels and renal interstitial fibrosis (99). Subsequent clinical trials in T2DM patients have confirmed its effect on lowering blood glucose and glycated hemoglobin and alleviating liver and kidney damage (100). After the application of Tang Shen Formula in patients with early DKD, microalbuminuria and macroalbuminuria were reduced, and renal function was improved. The efficacy and safety of this formulation are well demonstrated in clinical application (101). As for herbs and extracts, they can restore the intestinal immune barrier, inhibit inflammation and oxidative stress, and reduce the production of uremic toxins to realize the protective mechanism of kidneys by regulating gut microflora. Chinese herbs, due to their low-risk characteristics, provide novel and extensive drug choices for the management of DKD.

### 5.4 Fecal microbiota transplantation

Fecal microbiota transplantation (FMT) is a therapeutic method of transplanting the functional microbiota of a healthy donor into the gastrointestinal tract of a patient, often used in intestinal diseases and metabolic syndrome (102). In the animal experiment stage, the serum acetate level of diabetic rats taking FMT in the healthy group was decreased, insulin sensitivity was improved, weight was reduced, and renal interstitial inflammation injury and glomerular filtration membrane injury were improved (103). The safety of FMT has been suggested in clinical trials for the treatment of ulcerative colitis and obesity (104, 105). At present, the exploration of FMT in DN diseases mostly stays in animal experiments, but since it is generally believed that FMT is relatively safe, FMT is also a potential research direction for the treatment of DN.

### 5.5 Other therapeutic options for modulating the gut microbiota in DKD

At present, it can be seen that the application of broad-spectrum antibiotics can inhibit most of the gut microflora, inhibit the activation of the renal RAS system, reduce the level of inflammation, and kidney damage and decrease renal tubulointerstitial cholesterol in DN rats (106). Antibiotics are widely used in the treatment of kidney diseases, but the abuse of antibiotics may also lead to an imbalance of intestinal homeostasis and aggravate the damage to the kidney (107). In addition, as diabetes is a disease closely related to lifestyle, increasing exercise and controlling diet are beneficial to human health. In recent studies, exercise training as a non-pharmacological treatment method has been found to promote the recovery of gut microecology of diabetic mice, restoring the number of Firmicutes and Bacteroides, and reshaping the intestinal immune barrier of mice (108, 109). In clinical trials, the intestinal barrier of T2DM patients recovered during exercise for up to 6 months, and the inflammatory indicators of the body decreased significantly (110). Unfortunately, the current application of exercise therapy is more limited to the application of diabetes patients, but its regulatory effect on gut microbiota has led researchers to speculate about its alleviating effect on DN.

## 6 Conclusion

DKD is one of the important complications of diabetic microvascular injury. DKD poses a great threat to human health because of its concealment and high prevalence. In the process of exploring its pathogenesis and seeking treatment methods, researchers have found that DKD is closely related to the disorder of gut microbiota. Gut microbiota is an important factor affecting human metabolism, which participates in the daily operation of many organs such as heart, brain, lung and so on. Disordered gut microbiota produces large amounts of LPS, gut derived toxins, and affects bile acid metabolism, leading to intestinal inflammatory environment and intestinal epithelial barrier destruction. Toxins enter the blood circulation, and bacterial microflora shift, leading to a state of chronic low-grade inflammation. The accumulation of harmful metabolites in the kidney mediates the increase of renal inflammatory factors, glomerular filtration membrane damage, and renal tubulointerstitial fibrosis by activating signaling pathways. Finally, DKD patients showed increased proteinuria, decreased GFR, and other manifestations of renal insufficiency. A decrease in protective effect accompanies the increase of intestinal destructive factors in DKD patients. The increase of intestinal destructive factors in DKD patients is accompanied by a decrease in protective effect. SCFA acts on the kidney to reduce proteinuria, stabilize glomerular basement membrane podocytes, and reduce glomerulosclerosis and renal inflammation. Reducing SCFA-producing bacteria leads to intestinal anti-inflammatory and immunomodulatory dysfunction, and impaired glucose control. Due to the accumulation of metabolites after intestinal flora disorder, researchers have tried to find specific metabolites or metabolic pathways for early diagnosis and treatment of DKD by sequencing and metabolomics. Early intervention can improve the adverse renal outcomes, reduce the occurrence of cardiovascular complications, and prolong the life cycle of patients. At present, the treatment of DKD mainly adopts symptomatic treatment, such as reducing proteinuria and controlling diabetes. Modulating the gut microbiota to control blood sugar and alleviate the damage of kidney is an emerging treatment options. Oral drugs such as prebiotics, probiotics, and herbs have shown outstanding therapeutic effects on anti-inflammation, regulating intestinal microbiota, and protecting the kidney in clinical application. New therapies such as FMT and exercise training therapy have shown excellent effects on intestinal microbiota regulation and insulin resistance in diabetes mellitus, but they still lack clinical application in diabetic patients with kidney injury. For patients with DKD, delaying the progression of kidney disease and reducing cardiovascular complications are treatment goals. Delaying the progression of DKD by regulating intestinal flora is an important method for the prevention and treatment of DKD in the future.
