# Corrigendum: Research progress of gut microbiome and diabetic nephropathy

**DOI:** 10.3389/fmed.2024.1549438

**Published:** 2025-01-08

**Authors:** Chenling Chu, Tapas Ranjan Behera, Ying Huang, Wenhui Qiu, Jiayi Chen, Quanquan Shen

**Affiliations:** ^1^Department of Clinical Medicine, Hangzhou Normal University, Hangzhou, China; ^2^Department of Cancer Biology, Cleveland Clinic, Cleveland, OH, United States; ^3^Department of Public Health and Preventive Medicine, Hangzhou Medical College, Hangzhou, China; ^4^Department of Basic Medicine and Forensic Medicine, Hangzhou Medical College, Hangzhou, China; ^5^Department of Nephrology, Zhejiang Provincial People's Hospital Bijie Hospital, Bijie, China; ^6^Department of Nephrology, Urology & Nephrology Center, Zhejiang Provincial People's Hospital (Affiliated People's Hospital, Hangzhou Medical College), Hangzhou, China

**Keywords:** diabetic nephropathy, gut microbiota, short-chain fatty acids, inflammation, enteric-renal axis, probiotics

In the published article, there was an error in affiliations [2, 3, 4, 5, 6]. Instead of “[Chenling Chu^1,2^, Tapas Ranjan Behera^3^, Ying Huang^2,4^, Wenhui Qiu^2,5^, Jiayi Chen^1,2^ and Quanquan Shen^2,6*^

^1^Department of Clinical Medicine, Hangzhou Normal University, Hangzhou, China

^2^Department of Nephrology, Urology & Nephrology Center, Zhejiang Provincial People's Hospital (Affiliated People's Hospital, Hangzhou Medical College), Hangzhou, China

^3^Department of Cancer Biology, Cleveland Clinic, Cleveland, OH, United States

^4^Department of Public Health and Preventive Medicine, Hangzhou Medical College, Hangzhou, China

^5^Department of Basic Medicine and Forensic Medicine, Hangzhou Medical College, Hangzhou, China

^6^Department of Nephrology, Zhejiang Provincial People's Hospital Bijie Hospital, Bijie, China],” it should be “[Chenling Chu^1^, Tapas Ranjan Behera^2^, Ying Huang^3^, Wenhui Qiu^4^, Jiayi Chen^1^ and Quanquan Shen^5,6*^

^1^Department of Clinical Medicine, Hangzhou Normal University, Hangzhou, China

^2^Department of Cancer Biology, Cleveland Clinic, Cleveland, OH, United States

^3^Department of Public Health and Preventive Medicine, Hangzhou Medical College, Hangzhou, China

^4^Department of Basic Medicine and Forensic Medicine, Hangzhou Medical College, Hangzhou, China

^5^Department of Nephrology, Zhejiang Provincial People's Hospital Bijie Hospital, Bijie, China

^6^Department of Nephrology, Urology & Nephrology Center, Zhejiang Provincial People's Hospital (Affiliated People's Hospital, Hangzhou Medical College), Hangzhou, China].”

The authors apologize for this error and state that this does not change the scientific conclusions of the article in any way. The original article has been updated.

